# Construction of a knowledge graph for breast cancer diagnosis based on Chinese electronic medical records: development and usability study

**DOI:** 10.1186/s12911-023-02322-0

**Published:** 2023-10-10

**Authors:** Xiaolong Li, Shuifa Sun, Tinglong Tang, Ji Lu, Lijuan Zhang, Jie Yin, Qian Geng, Yirong Wu

**Affiliations:** 1 Yichang Key Laboratory of Intelligent Medicine, Yichang, Hubei China; 2 College of Economics and Management, China Three Gorges University, Yichang, Hubei China; 3 College of Computer and Information Technology, China Three Gorges University, Yichang, Hubei China; 4 Yichang Central People’s Hospital, Yichang, Hubei China; 5 The Fourth Affiliated Hospital of Nanjing Medical University (Pukou Hospital), Nanjing, Jiangsu China; 6 Institute of Advanced Studies in Humanities and Social Sciences, Beijing Normal University, No18 Jinfeng Road, Tangjiawan, Xiangzhou District, Zhuhai, 519087 Guangdong China

**Keywords:** Chinese electronic medical records, Breast cancer, Knowledge graph, Mammography, Computer assisted diagnosis

## Abstract

**Background:**

Electronic medical records (EMRs) contain a wealth of information related to breast cancer diagnosis and treatment. Extracting relevant features from these medical records and constructing a knowledge graph can significantly contribute to an efficient data analysis and decision support system for breast cancer diagnosis.

**Methods:**

An approach was proposed to develop a workflow for effectively extracting breast cancer-related features from Chinese breast cancer mammography reports and constructing a knowledge graph for breast cancer diagnosis. Firstly, the concept layer of the knowledge graph for breast cancer diagnosis was constructed based on breast cancer diagnosis and treatment guidelines, along with insights from clinical experts. .Next, a BiLSTM-Highway-CRF model was designed to extract the mammography features, which formed the data layer of the knowledge graph. Finally, the knowledge graph was constructed by combining the concept layer and the data layer in a Neo4j graph data platform, and then applied in visualization analysis, semantic query and computer assisted diagnosis.

**Results:**

Mammographic features were extracted from a total of 1171 mammography examination reports. The overall extraction performance of the model achieved an accuracy rate of 97.16%, a recall rate of 98.06%, and a F1 score of 97.61%. Additionally, 47,660 relationships between entities were identified based on the four different types of relationships defined in the concept layer. The knowledge graph for breast cancer diagnosis was constructed after inputting mammographic features and relationships into the Neo4j graph data platform. The model was assessed from the concept layer, data layer, and application layer perspectives, and showed promising results.

**Conclusions:**

The proposed workflow is applicable for constructing knowledge graphs for breast cancer diagnosis based on Chinese EMRs. This study serves as a reference for the rapid design, construction, and application of knowledge graphs for diagnosis and treatment of other diseases. Furthermore, it offers a potential solution to address the issues of limited data sharing and format inconsistencies present in Chinese EMR data.

## Background

Breast cancer poses a significant threat to women’s health worldwide and is one of the most prevalent malignant tumors among women. Currently, the diagnosis and treatment of breast cancer heavily rely on experienced clinical experts. However, the scarcity of clinical experts and their overwhelming workload severely impact timely diagnosis and treatment for patients. Although certain deep learning algorithms can be used to detect breast cancer to some extent, their lack of interpretability remains a major technical barrier, preventing their practical application in clinical settings [1]. Advances in science and technology have significantly improved the accuracy of breast cancer diagnosis and treatments. Consequently, a vast amount of breast cancer examination data has been generated and stored in the Electronic Medical Record (EMR) system, containing valuable information about the disease. Extracted variables from EMRs can contribute to patients’ condition management [2] and aid in breast cancer risk prediction [3]. Moreover, these variables can serve as knowledge-driven tools, enhancing diagnosis efficiency and interpretability, ultimately reducing errors and relieving clinician workloads [4]. Therefore, there is an urgent need to design an effective data processing and management system to help collect, manage, and use variables for breast cancer diagnosis and treatments.

Knowledge graph (KG) is one of the visualization technologies in the knowledge domain. It is composed of entities, semantic types, properties, and relationships [5]. In medical domains, research on the construction of knowledge graph has achieved many successes [6, 7]. However, most of the existing knowledge graphs in medical domains are based on data from medical literature, online community resources, or various open databases that are publicly available. Although this type of data is more convenient to obtain, it is not real-world EMR data [8]. Additionally, the quality of knowledge graphs does not meet the needs of disease diagnosis or treatment. Particularly, the Chinese EMR data has poor sharing characteristics, diverse storage approaches, different formats and standards, etc., making it difficult to build a knowledge graph [9]. Therefore, it is necessary to develop a workflow for breast cancer KG construction using Chinese EMR data.

For studies on breast cancer knowledge graph construction, An [10] proposed a method to extract features about breast cancer from EMRs to construct the knowledge graph for breast cancer diagnosis. The design of the concept layer and the data layer was specifically introduced. However, a description of the procedure used for knowledge graph construction is missing, which makes it impossible to check the effect of the constructed knowledge graph. Hasan et al. [11, 12] proposed a method to construct a knowledge graph from cancer registration data and developed a prototype of the knowledge graph for breast cancer patient management. However, information contained in the knowledge graph is very limited for breast cancer diagnosis. Jin et al. [13] proposed a method to construct knowledge graphs for breast cancer diagnosis and treatments. Nevertheless, a description of the construction procedure is missing and the construction results are not reported, affecting the evaluation of the construction method. There are some issues with existing methods for constructing breast cancer knowledge graphs from Chinese EMRs. Firstly, existing methods exhibit limited coverage in building breast cancer knowledge graphs, failing to encompass a comprehensive range of knowledge. Secondly, while some existing methods can be used to construct knowledge graphs, most of them lack sufficient detail and clarity when the construction process and methodology are described. Thirdly, these methods have not been validated through clinical applications.

Commonly used examination methods for breast cancer include clinical palpation, mammography, ultrasound, and nuclear magnetic resonance. Mammography is currently one of the most widely used diagnostic method for breast cancer. Using breast cancer mammography examination reports as research samples, case studies can be conducted on knowledge extraction, knowledge graph construction, and applications, which can help obtain standardized and high-quality examination results to facilitate effective disease management. Additionally, breast cancer risk prediction can be performed to assist in breast cancer diagnostic decision-making. However, there is still a scarcity of research in this domain. Therefore, it is necessary to develop a comprehensive workflow, from knowledge extraction from Chinese EMRs to knowledge graph construction, and even diagnosis applications.

Our study intends to utilize Named Entity Recognition (NER) technology to effectively identify and extract features from Chinese breast cancer mammography reports and construct a knowledge graph for breast cancer diagnosis. The contributions of this study are as follows:

- A workflow covering the design of the concept layer, feature extraction from Chinese EMRs, the construction of a knowledge graph, and a demonstration of its applications is proposed. Based on the workflow, a top-down knowledge graph for breast cancer diagnosis is constructed.
- A deep learning model is developed, the BiLSTM-Highway-CRF network, which achieves higher extraction performance compared to traditional models for feature extraction from Chinese EMRs.
- The constructed knowledge graph is utilized to realize visual analysis, semantic query, and computer-assisted diagnosis, effectively demonstrating its usefulness and practicality in clinical applications.

## Methods

The approval was obtained from the hospital’s ethics review committee for this study. There are 2989 mammography examination reports collected from the Radiology department of a Three-A hospital located in Yichang province of China, spanning from December 2018 to July 2019. The data were then subjected to de-identification processing. Each report was prepared by one doctor and verified by another doctor. A total of nine doctors participated in this work. After duplicated reports were removed and reports with incomplete or incorrect data were deleted, the final dataset consists of 1171 reports used to construct the knowledge graph for breast cancer diagnosis.

**Fig. 1:**
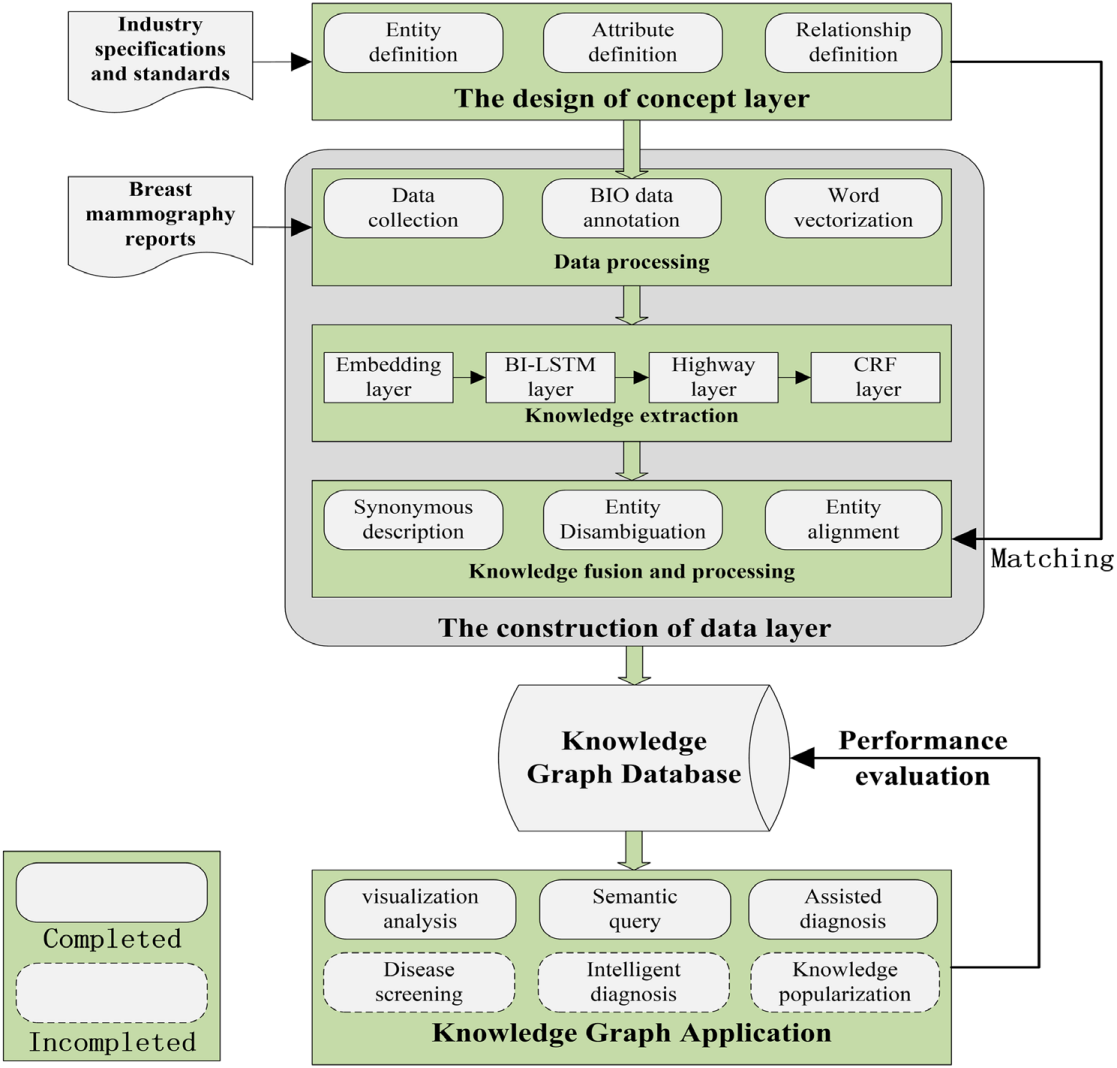
The workflow of constructing a knowledge graph for breast cancer diagnosis based on mammography examination reports

The knowledge graph construction process is shown in Fig. 1, which mainly includes four steps: (1) the design of the concept layer. (2) the development of the data layer, which is composed of data annotation, feature extraction, and knowledge fusion. (3) knowledge graph construction. (4) knowledge graph applications. The details of each step are described in the following subsections.

### Design of the concept layer

**Fig. 2:**
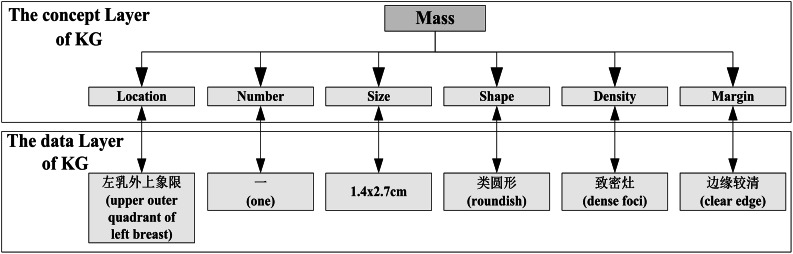
The relationship between the concept layer and the data layer of a knowledge graph

A knowledge graph is composed of a concept layer (or schema layer) and a data layer (or instance layer) [14], as shown in Fig. 2. The concept layer is the core of the knowledge graph, including a hierarchical structure of entities and their attributes, which can be used to constrain data storage in the data layer [15].

In the design of the concept layer, we leveraged the Breast Imaging Reporting and Data System (BI-RADS) lexicon of the American College of Radiology [16], and the Breast Cancer Diagnosis and Treatment Guidelines issued by the Chinese Anti-Cancer Association [17]. Additionally, we engaged four mammography radiologists from different hospitals to participate in ontology definition and concept layer framework design. Moreover, we extensively referenced domestic and international literature, as well as mammography examination norms and standards, to revise the concept layer .

The concept layer is designed as a 3-level hierarchical structure with 15 types of mammography features primarily(Calcification, Density, Distribute, Location, Mass, Lymph Node, Margin, Merge, Number, Shape, Size, Special, Structure, Category descriptions, Negation). There are 4 entities such as “common signs” at the first level, there are 9 entities such as “Mass” at the second level, and related entity attributes are at the third level (Table 1). According to the three-element principle of knowledge graph construction [18], it is necessary to clarify the three elements of entity-relationship-entity attribute or entity-relationship-entity. Guided by clinicians, we established relationships among entities and attributes. This study not only defined a set of entities and their attributes, but also established their hierarchical relationships, Table 2 shows the three elements of subject, predicate and object among different entities and attributes in the concept layer. Once entities, entity attributes, and relationships were specified, the design of the concept layer of the knowledge graph for breast cancer diagnosis was completed. In the following context, the term “entity” refers to mammography examination variable such as “mass” and “calcification”, while “entity attribute” refers to attribute variables such as “size” and “shape” owned by “entity”. Both examination variables and attribute variables belong to BI-RADS variables.

**Table 1:** The entities and attributes related to mammography features in the concept layer

Project	First-level entity	Second-level entity	Entity attributes
Breast cancer mammography	Common signs	Mass	Location, Margin, Number, Shape, Type, Size, Density, Negation,
		Calcification	Location, Number, Shape, Type, Size, Density, Negation, Distribute
		Structure	Describe
	Special signs	Duct change	Describe
		Intramammary lymph nodes	Location, Number, Shape, Negation, Describe
		Asymmetrical	Location, Describe
	Merge signs	Vascular change	Location, Shape, Negation,
		Axillary lymph nodes	Location, Number, Shape, Negation, Describe
		Association anomaly	Location, Number, Negation, Describe
	Category descriptions		Location, Describe

**Table 2:** Triple-structure of the subject, predicate, and object in the concept layer

	subject	predicate	object
1	Entity (Basic information)	select	Attributes (Name, Sex, etc.)
2	Entity (Report)	has_a	Entity (Basic information, Mammography information, BI-RADS information)
3	Entity (Mammography information)	instance_of	First-level entity (Common signs, Merge signs, etc.)
4	First-level entity (Common signs, Merge signs, etc.)	part_of	Second-level entity (Mass, Calcification, Structure, etc.)
5	Second-level entity (Structure, Mass, etc.)	select	Attributes (Location, Size, etc.)

### Development of the data layer

#### Data annotation

In this study, entity type annotation was performed on mammography examination reports for developing entity recognition models. The annotation step was carried out using the web-based labeling tool BRAT [19]. Then, the annotation text was prepared in the BIO format (“B” indicates the start tag of an entity, “I” indicates the tag inside an entity, and “O” indicates the tag outside an entity) [20], commonly used in NER tasks. Finally, the Word2Vec model was used for word vectorization [21].

#### Feature extraction

There are three types of NER methods that can be used to extract BI-RADS variables from EMRs: rule-based methods [22, 23], machine learning-based methods [24–26], and deep learning-based methods [27–29]. The first type of methods pays more attention to the doctor’s clinical experience, while the second and third type of methods rely more on data to train the models. Rule-based NER methods identify entities based primarily on hand-crafted semantic and syntactic rules, and have high development costs in terms of time and efforts from domain experts. Therefore, there exist the disadvantages such as poor portability, the problems of expanding to other entity types or datasets, and the difficulty of migrating to other fields [30]. Although machine learning-based methods have overcome some of these shortcomings, with the increase of data volume, efficiency and effectiveness may become issues. On the other hand, deep learning-based methods are suitable for nonlinear transformation, and they can handle the linguistic and structural variability of free text more effectively, which can be used to build a more complex network [22].

The BiLSTM-CRF model is currently one of the most popular NER models [31]. In this study, a BiLSTM-Highway-CRF model was constructed, comprising a BiLSTM module, a Highway network module, and a CRF module. The Highway network defines two gate structures, transfer gate $\text{T}({\text{W}}_{\text{T}},\text{x})$ and carry gate $\text{C}({\text{W}}_{\text{C}},\text{x})$, to control information flow. The transfer gate controls the amount of information transmitted by the feed forward network, while the carry gate controls the amount of information transmitted by the input x. Its mathematical expression is defined in Eq. (1), the highway network also sets the relationship between the transfer gate $\text{T}$ and the carry gate C, that is, the above mathematical expression can be simplified as Eq. (2). The design of the relationship between switching gates makes the Highway network more flexible.

1 $$y={\text{H}}\left( {{{\text{W}}_{\text{H}}},{\text{x}}} \right)*{\text{T}}\left( {{{\text{W}}_{\text{T}}},{\text{x}}} \right)+{\text{x*C}}\left( {{{\text{W}}_{\text{C}}},{\text{x}}} \right)$$

2 $$y={\text{H}}\left( {{{\text{W}}_{\text{H}}},{\text{x}}} \right)*{\text{T}}\left( {{{\text{W}}_{\text{T}}},{\text{x}}} \right)+{\text{x*}}\left( {1 - {\text{T}}\left( {{{\text{W}}_{\text{T}}},{\text{x}}} \right)} \right)$$

**Fig. 3:**
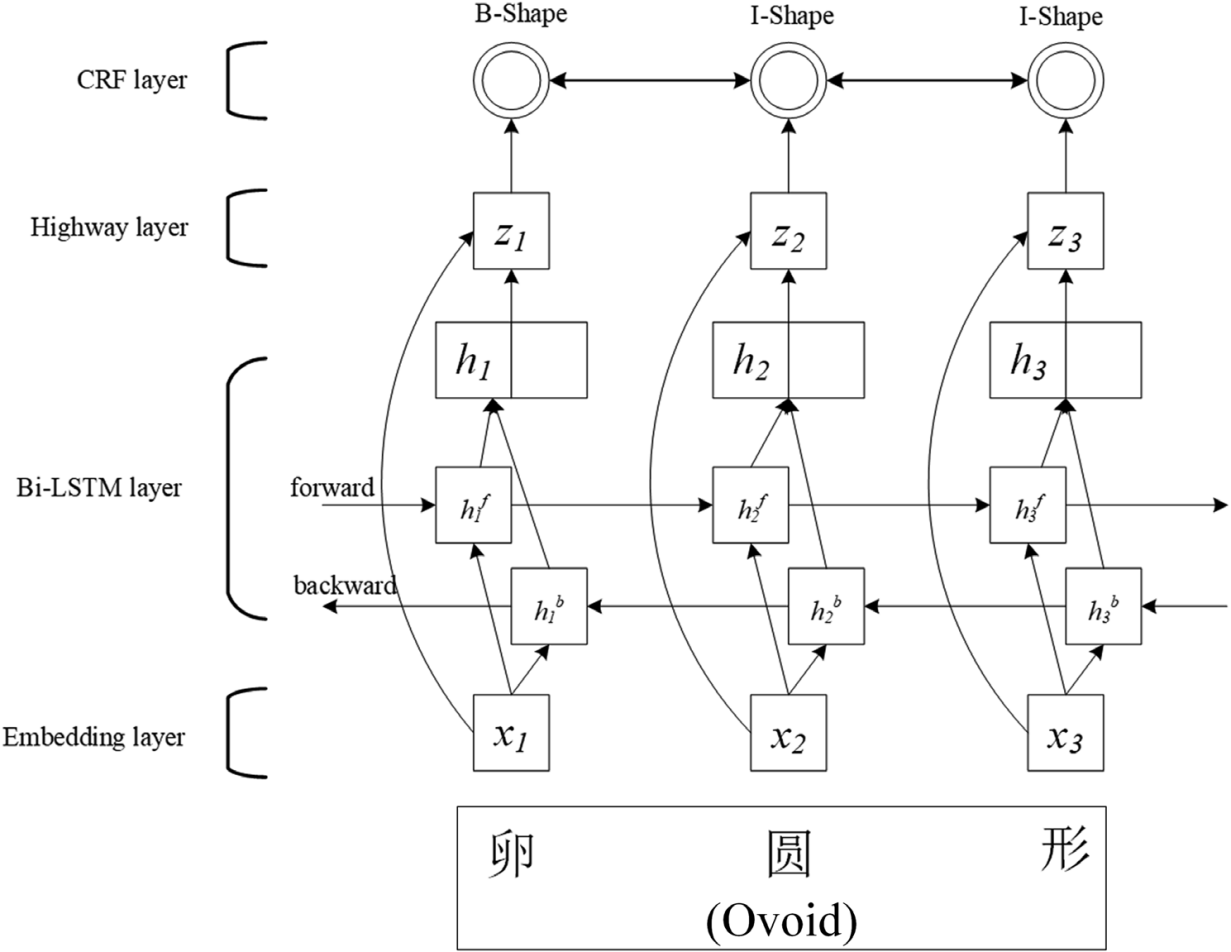
BiLSTM-Highway-CRF model

Based on the idea of the Highway network [32], through comparing the feature information obtained by the training of the BiLSTM network with the original word vector information, the retention ratio of the trained feature information and the word vector information can be optimized, so as to reduce the degradation impacts of the deep neural network on recognition performance. Figure 3 illustrates the structure of the BiLSTM-Highway-CRF model. “卵圆形”(Ovoid) is an input Chinese word. B-Shape, I-Shape, and I-Shape correspond to their recognition results.

In this study, the recognition performance of the model was evaluated using commonly used metrics in NER tasks, which include precision rate $P$, recall rate $R$, and $F1$ score. $P$ represents the proportion of real positive samples among the entities recognized by the model to be positive. $R$ represents the proportion of all positive samples that are correctly recognized. The $F1$ score is the harmonic average of precision and recall. The score of $F1$ is 1 (perfect precision and recall) to reach the best value, and 0 to reach the worst value.

#### Knowledge fusion and processing

During the process of extracting entities, we observed that the difference between clinician’s reporting habits and hospital operation guidelines results in different qualities of mammography reports. The same entity may be inconsistent in the context of different mammography reports. As a result, the extracted entities will be different. Based on breast cancer diagnosis and treatment guidelines, regulations, and industry standards [16, 17], we performed text similarity analysis [33] on the extracted mammography features of the same type to achieve the effects of synonymous descriptions. Additionally, we performed standard semantic replacements for words having semantic errors and synonymous descriptions to attain the goal of entity disambiguation. In the process of coreference resolution, we supplemented and aligned features to ensure the consistency and accuracy of the extracted mammography features.

### Knowledge graph construction and application

There are two main approaches for knowledge graph construction: bottom-up and top-down [34]. The bottom-up approach involves obtaining entities and frameworks directly from the data. However, due to its automated nature, the knowledge obtained through this approach may lack completeness and its quality may not reach a practical level. On the other hand, the top-down approach is mainly used to construct domain-specific knowledge graphs. For this approach, the construction process begins with domain experts and clinical professionals who define the entities and frameworks at the concept layer based on the needs of breast cancer diagnosis. The data is then filled into the knowledge graph from heterogeneous data sources.

To ensure that the knowledge graph is effectively utilized for clinical applications, especially in assisting diagnosis, this study adopted the top-down approach for knowledge graph construction. At first, the entities and frameworks at the concept layer were specified, and then data annotation was carried out with the assistance of clinical experts and doctors. Next, deep learning models were used for knowledge extraction from the annotated data. The extracted knowledge was then subjected to knowledge fusion, which involved standardizing the entities and entity attributes in the data layer. Afterward, the fused data layer was processed and matched with the concept layer, establishing relationships between entities and between entities and attributes to develop a large-scale hierarchical graph system. Finally, the knowledge graph construction was completed.

Due to the top-down method used to construct knowledge graph, all entities, entity attributes and relationships were defined in the concept layer [34]. Relying on the structure of the concept layer, the entities and entity attributes extracted from mammography reports were matched with the entities and attributes of the concept layer, so as to construct a complete knowledge graph. In order to facilitate the top-down application research, this study used Neo4j graph data platform (hereinafter referred to as Neo4j) to store knowledge in the form of graphic database. The high quality of knowledge graphs is required in the medical domain [35]. In order to verify the practicability and rationality of the knowledge graph, this study carried out experiments such as visual analysis, semantic query and computer-aided diagnosis using the knowledge graph.

## Results

### Annotation results

After the 1171 mammography examination reports were annotated with those 15 types of mammography features, the number of entities in each type was obtained (Table 3). Four breast cancer mammography examination doctors were invited to participate in and guide data annotation. After data annotation was completed, clinical doctors conducted multiple rounds of sampling and corrected any incorrectly annotated data. In the final round of sampling, 951 labeled data points from 50 data samples were inspected, and the accuracy of the annotated data reached 99.26%.

**Table 3:** The type and number of its entities annotated in this study

Entity type	Definition	Number of entities
Calcification	Calcification	1824
Density	Mass density, Tissue density	630
Distribute	Calcification distribution	144
Location	Description area, location information, like: upper limit on the left	6038
Mass	Mass information	1169
Lymph Node	Lymph Node information	1163
Margin	Mass boundary information	507
Merge	Merge signs. Sunken skin, Syndromes, such as thickening	1154
Number	The number of mass or calcification	1082
Shape	The shape of a mass or calcification	1270
Size	The size of mass or calcification	562
Special	Special signs, like: Vascular thickening	1217
Structure	Normal or distorted structure	1934
Category descriptions	The typing features about breast densities	1162
Negation	Negative Words	3288

### The results of feature extraction

For 1171 text reports, we used the featured extracted from 820 reports in the training set for model training, the features from 116 reports in the validation set for model parameter adjustment, and the features from 235 reports in the test set for evaluating the performance of the model.

The results of the BiLSTM-Highway-CRF model for each mammography feature type are described in Table 4. To compare the model performance, we also developed three widely used NER models: Hidden Markov Model (HMM), Conditional Random Field model (CRF), and BiLSTM-CRF. The results of the precision, recall, and F1 of those three NER models and the BiLSTM-Highway-CRF model are shown in Table 5. For the BiLSTM-Highway-CRF model, the precision rate is 97.16%, the recall rate is 98.06%, and F1 is 97.61. The experimental results show that the performance of the CRF model is higher than that of the HMM model in NER task, indicating that considering the proximity label information is essential for predicting the current label when extracting BI-RADS features from mammography examination reports. Additionally, the BiLSTM-Highway-CRF model demonstrates a higher performance than the CRF and BiLSTM-CRF models, which indicates that the introduction of the highway network mechanism is beneficial for the model to learn features in mammography examination reports.

**Table 4:** Performance of BiLSTM-Highway-CRF model for each entity type(unit: %)

Entity Type	Precision	Recall	F1
Calcifications	99.71	100.00	99.86
Density	91.59	93.33	92.45
Distribute	93.02	93.02	93.02
Location	97.42	97.89	97.65
Mass	98.71	99.57	99.13
Lymph Node	98.30	98.72	98.51
Margin	89.57	94.81	92.11
Merge	94.14	96.98	95.54
Number	97.96	97.56	97.76
Shape	93.57	96.04	94.79
Size	97.56	98.77	98.16
Special	97.47	97.88	97.67
Structure	98.16	98.42	98.29
Category descriptions	98.28	99.57	98.92
Negation	99.84	100.00	99.92

**Table 5:** Overall feature extraction performance(unit: %)

Model	Precision	Recall	F1
HMM	93.42	94.91	94.17
CRF	97.00	95.84	96.42
BiLSTM-CRF	96.71	97.20	96.96
BiLSTM-Highway-CRF	**97.16**	**98.06**	**97.61**

### The results of relationship matching

There are a total of 47,660 relationships between entities and between entities and attributes in 1,171 reports. Among them, there are 3,513 “*has_a*” relationships, 4,684 “*instance_of”* relationships, 17,565 “*part_of”* relationships and 21,898 “*select”* relationships.

### The applications of knowledge graph

#### Visualization analysis

**Fig. 4:**
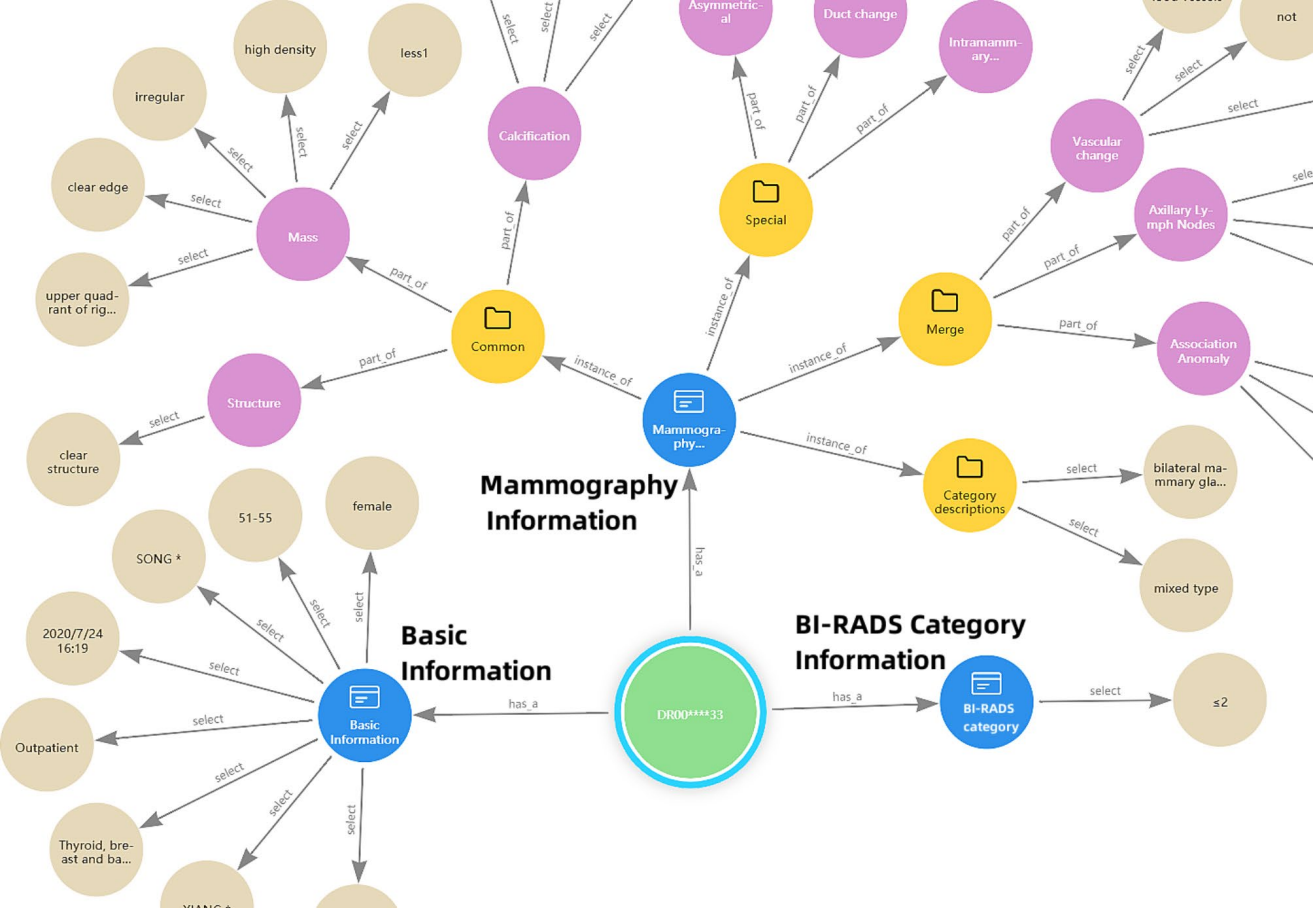
An example of the knowledge graph(part)

The knowledge graph for breast cancer diagnosis is constructed by combining the concept layer and the data layer. Additionally, the demographic patient information and BI-RADS categories representing the results of breast cancer diagnosis are integrated into the knowledge graph (Fig. 4). In the knowledge graph, nodes represent entities or entity attributes, and edges represents the relationships between entities or between entities and their attributes. This visualization makes it convenient for patients and clinicians to view and analyze patient situations.

#### Semantic query

**Fig. 5:**
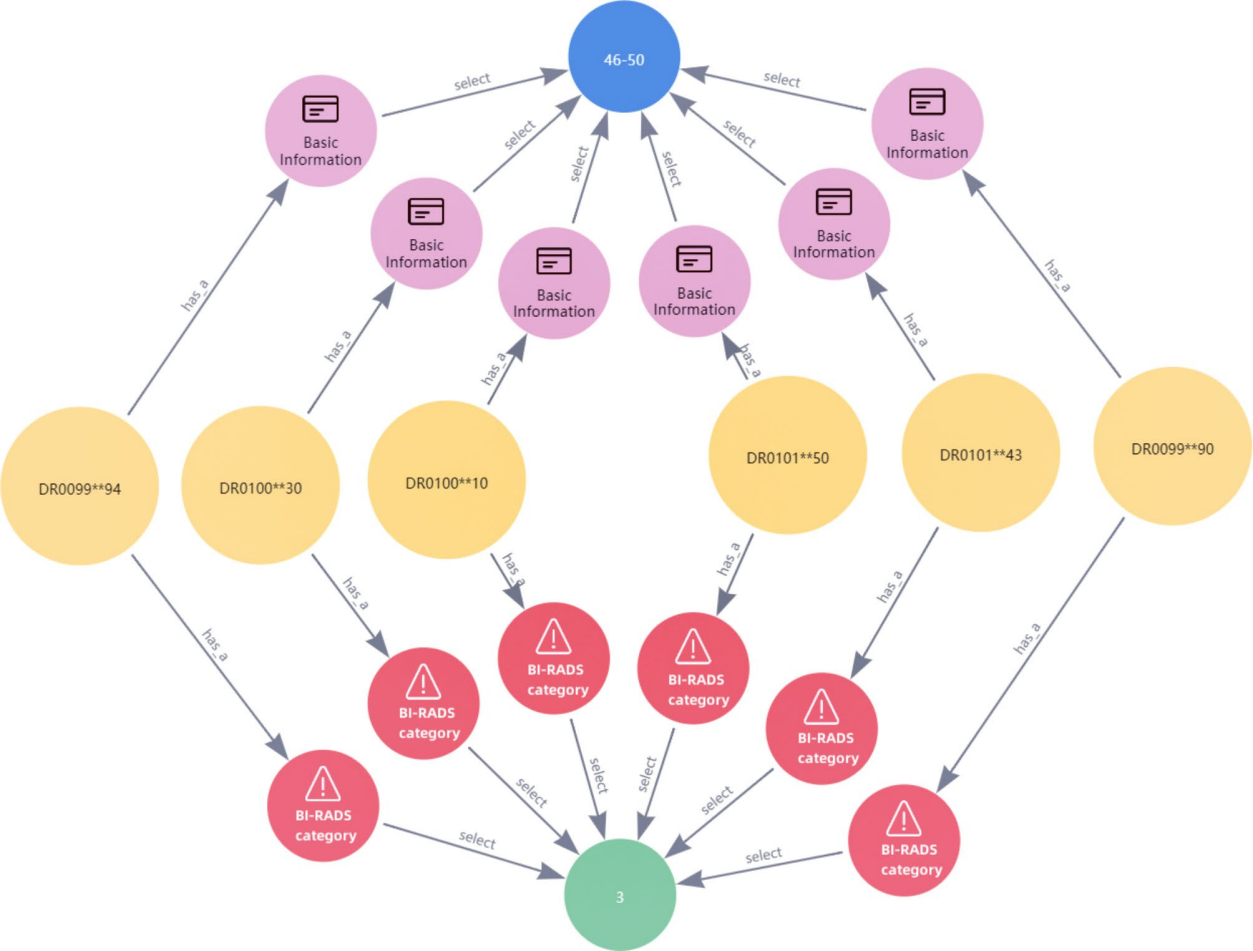
One example of customizing the query statements

To facilitate clinicians in accessing and analyzing data, we have customized several advanced query statements using the Neo4j graph data platform. For example, based on common queries required by clinicians, we have predefined template statements with parameters. By entering specific queries, such as “patient information between 46 and 50 and BI-RADS level 3,“ clinicians can retrieve the corresponding patient information (Fig. 5). Relying on the knowledge graph, clinicians can complete simple query, combined query, conditional query, path query and even deep relationship query of patient information without a need of learning a lot of non-medical professional knowledge.

#### Computer assisted diagnosis

**Fig. 6:**
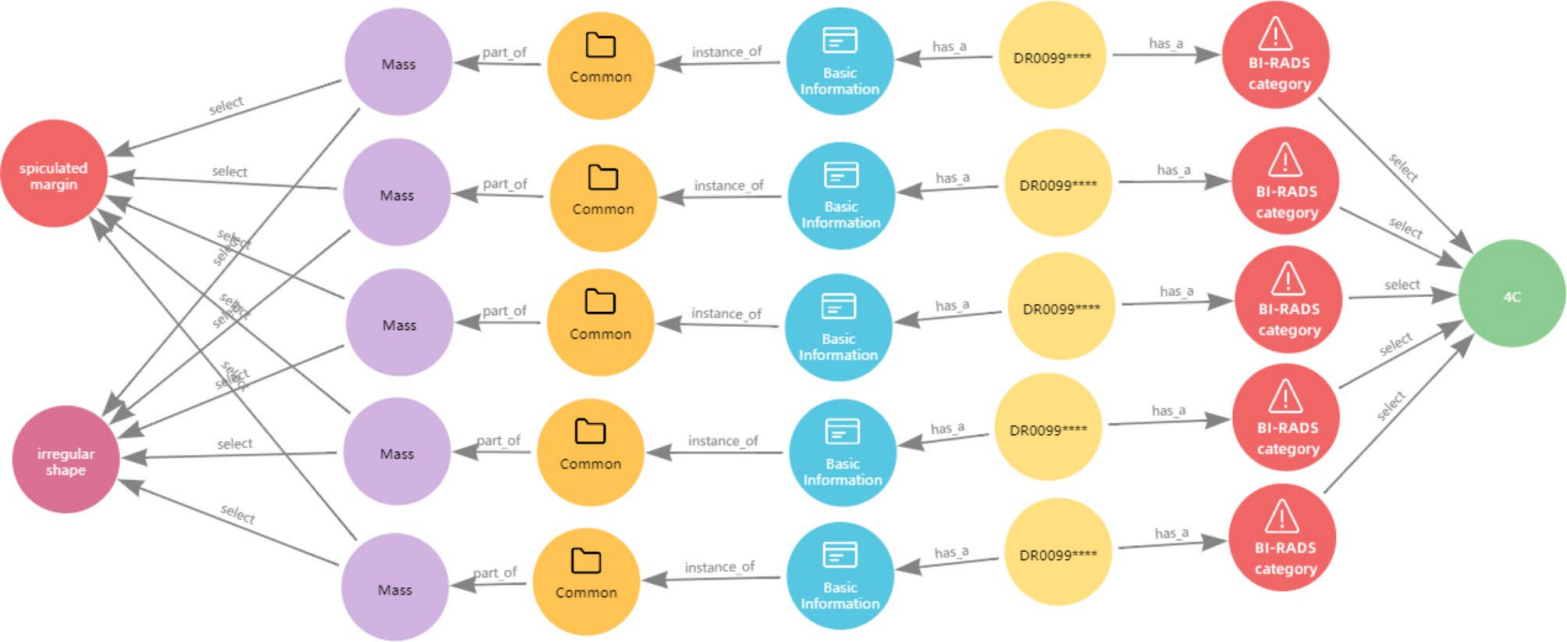
The relationship between the feature “irregular shape and spiculated margins” and BI-RADS category “4 C” in the knowledge graph

Breast cancer diagnosis involves numerous mammography features, making it essential to identify the most critical risk factors used for accurate diagnosis. Through analysis of the knowledge graph, we have discovered that for some patients who have the mass that is an irregular shape with spiculated margins, their examination result indicate BI-RADS category of 4 C (Fig. 6). A BI-RADS category of 4 C indicates a highly suspicion of malignancy, and a blurred margin is considered one of the highest risk factors. Clinical evaluation has confirmed the accuracy of this conclusion. By extension, we can assess the impact of individual other features or combinations of features on the BI-RADS classification.

Furthermore, the knowledge graph enables us to assess the impact of individual features or combinations of features on the BI-RADS classification. By leveraging the information from the knowledge graph, clinicians can gain valuable insights into the diagnostic process and utilize it to assist in the diagnosis of breast cancer more effectively. Overall, the knowledge graph constructed in this study has the potential to play a vital role in improving breast cancer diagnosis.

### Quality evaluation

To assess the quality of experimental data, model performance, and application effectiveness, we invited eight clinical experts and natural language processing experts to conduct testing, analysis, and data quality assessment at the concept layer, data layer, and application layer (as shown in Table 6). The specific evaluations are as follows:

**Table 6:** The indexes and results of effective evaluation

Knowledge graph	Test and Analysis Content	Data Quality Assurance and Evaluation	Suggestion
the concept layer	Ontology and attribute definition, relationship construction, concept layer framework	**Positive aspects**: Accuracy, rationality, completeness, standardization **Areas for improvement**: Generality, effectiveness	Further standardize and refine the concept layer framework of the knowledge graph to ensure its generality and effectiveness.
the data layer	Data preprocessing, knowledge extraction, knowledge fusion, knowledge processing	**Positive aspects**: Precision, accuracy, recall, F1 **Areas for improvement**: Robustness, portability	Conduct additional experiments on multi-source data to optimize the model and enhance its robustness and generalization capabilities.
the application layer	Visualization analysis, data queries, assisted diagnosis	**Positive aspects**: Visualization, structured presentation, accuracy, usability, user-friendliness **Areas for improvement**: Completeness, operability, aesthetics, practicality	Integrate other feature knowledge graphs (such as more detailed basic information, other breast cancer examination data, breast cancer malignancy information), and suggest utilizing machine learning or deep learning for knowledge inference to improve the clinical application of the model.

In the concept layer design, the ontology and concept layer design of the breast cancer mammography diagnosis knowledge graph were completed (as shown in Tables 1 and 2). The design includes 4 first-level entities, 9 s-level entities, and 39 entity attributes, encompassing common BI-RADS variables used in breast cancer mammography examinations from both domestic and international sources. This demonstrates the accuracy, rationality, completeness, and standardization of the knowledge graph framework. However, the top-down construction approach places high demands on the design of the concept layer. It is recommended to further standardize and refine the knowledge graph concept layer framework to ensure its generality and effectiveness, thus ensuring that the knowledge graph complies with clinical standards and meets clinical application requirements.

In the data layer design, the BiLSTM-Highway-CRF deep learning model for knowledge extraction significantly improved the precision, recall, and F1 score compared to the HMM, CRF, and BiLSTM-CRF models, achieving 97.16%, 98.06%, and 97.61%, respectively. This demonstrates the accuracy and precision of data extraction. However, since the model data all originate from the same hospital, its accuracy on data from other sources cannot be guaranteed. Further experiments on multi-source data are recommended to optimize the model and enhance its robustness and generalization capabilities.

In the application layer, the knowledge graph is completed, which can be used to effectively present patient information, as indicated by visualization analysis and data queries. (as shown in Fig. 4). The retrieval results (as shown in Fig. 5) demonstrate accuracy, comprehensiveness, and usability of the knowledge graph. However, there is room for improvement in terms of user-interface. Additionally, the knowledge graph can provide a theoretical basis and practical foundation for assisted diagnosis of breast tumor malignancy (as shown in Fig. 6). It is recommended to integrate other knowledge graphs as well as machine learning or deep learning algorithms for knowledge inference to expedite the clinical application of the knowledge graph.

## Discussion

Currently, the majority of widely used knowledge graphs in the medical domains are constructed using the bottom-up approach [36], where the data is generally obtained from medical literature, online community resources, or various open databases. Although this kind of data is easier to obtain, it does not represent real-world EMR data. Additionally, the quality of knowledge graphs constructed using this approach may not meet the requirements for disease diagnosis or treatment [37]. To address these challenges, we have not only designed the workflow which utilizes the top-down approach for knowledge graph construction, incorporating real-world EMR data, specific domain knowledge, and clinician’s experience, but we have also successfully constructed the knowledge graph for breast cancer diagnosis following this approach.

In this study, we have developed the BiLSTM-Highway-CRF model to effectively extract mammography features from mammography reports. The performance of the BiLSTM-Highway-CRF model outperforms that of both the CRF model and the BiLSTM-CRF model, indicating that the incorporation of the highway network mechanism is advantageous for the model to learn features in examination reports more accurately and comprehensively. The extracted BI-RADS variables from breast cancer mammography reports are then integrated and organized within the knowledge graph specifically designed for breast cancer diagnosis. By leveraging the graphic structure of the knowledge graph, we have established an efficient data visualization and management approach. Overall, the successful development and application of the BiLSTM-Highway-CRF model and the knowledge graph demonstrate the potential of these techniques in advancing breast cancer diagnosis and management.

## Conclusions

The main contribution of this study lies in the development of a comprehensive workflow that encompasses the extraction of Chinese EMR variables and the application of knowledge graphs, providing guidance for the construction and utilizing medical knowledge graphs in disease diagnosis and treatment. In this study, we design the concept layer of the knowledge graph with reference to BI-RADS standards and several breast cancer diagnosis and treatment guidelines. Based on the deep learning NLP methods, mammographic features are extracted from examination reports and imported into the Neo4j graph data platform. Leveraging the design of the concept layer, we develop the knowledge graph for breast cancer diagnosis. Through the evaluation of the design of the concept layer, the construction of the data layer, and the functions of the application layer, the rationality, effectiveness, and practicability of the knowledge graph are demonstrated.

In conclusion, this study provides a valuable workflow that serves as a guide for designing, constructing, and applying knowledge graphs in the diagnosis and treatment of breast cancer. Moreover, it offers insights for designing and constructing knowledge graphs for other disease diagnosis and treatment scenarios. To a certain extent, it contributes to addressing issues related to poor data sharing and format inconsistencies in Chinese EMR data. For future research, we aim to emphasize the construction of the concept layer in the knowledge graph to enhance its effectiveness and generalizability. Additionally, we will utilize larger datasets from multiple hospitals to further enrich and develop the knowledge graph, thereby improving the model’s robustness. Lastly, ethical considerations will be considered when the knowledge graph is implemented in clinical disease diagnosis applications.

## Data Availability

This research made use of the data of EMR from Yichang Central People’s Hospital. The data can be made available upon reasonable request from the corresponding author. The data are not publicly available due to privacy restrictions. The source codes of this research are available at, https://github.com/hbxiaolong/NER-KG.

## Abbreviations

NLP: Natural Language Processing
EMR: Electronic Medical Record
KG: Knowledge Graph
BI-RADS: Breast Imaging-Reporting And Data System
CRF: Conditional Random Fields
BiLSTM: Bi-directional Long Short-Term Memory
RDF: Resource Description Framework
